# Squatting-Related Tibiofemoral Shear Reaction Forces and a Biomechanical Rationale for Femoral Component Loosening

**DOI:** 10.1155/2014/785175

**Published:** 2014-05-20

**Authors:** Ashvin Thambyah, Justin Fernandez

**Affiliations:** ^1^Department of Chemical and Materials Engineering, University of Auckland, 20 Symonds Street, Auckland 1142, New Zealand; ^2^Auckland Bioengineering Institute, University of Auckland, 70 Symonds Street, Auckland 1010, New Zealand; ^3^Department of Engineering Science, University of Auckland, 70 Symonds Street, Auckland 1010, New Zealand

## Abstract

Previous gait studies on squatting have described a rapid reversal in the direction of the tibiofemoral joint shear reaction force when going into a full weight-bearing deep knee flexion squat. The effects of such a shear reversal have not been considered with regard to the loading demand on knee implants in patients whose activities of daily living require frequent squatting. In this paper, *the shear reversal effect* is discussed and simulated in a finite element knee implant-bone model, to evaluate the possible biomechanical significance of this effect on femoral component loosening of high flexion implants as reported in the literature. The analysis shows that one of the effects of the shear reversal was a switch between large compressive and large tensile principal strains, from knee extension to flexion, respectively, in the region of the anterior flange of the femoral component. Together with the known material limits of cement and bone, this large mismatch in strains as a function of knee position provides new insight into how and why knee implants may fail in patients who perform frequent squatting.

## 1. Introduction

There is still much to be done to improve the design of knee implants both in terms of longevity and their ability to serve a wider range of patient needs. One particular need involves improving knee implant design to satisfy populations requiring a postoperative ability to perform deep knee bending and squatting [1–3]. The urgency of this problem is exacerbated by the increasing medical needs of aging Asian populations [4], in which deep knee bending and squatting are common activities of daily living, presenting the call for better design in implants that will allow deep flexion to be performed safely and reliably, without affecting the expected longevity of the implant.

High flexion knee replacement strategies have included retaining the cruciate ligament, specific intraoperative soft tissue balancing, and specially designed knee implants that control femoral rollback, improve joint conformity in large flexion angles, or provide more freedom in axial rotation [5]. Despite these efforts, studies have reported relatively limited long-term success [6–9], with femoral component loosening being identified as one of the more common negative outcomes in high-flexion knee arthroplasty. Such femoral component loosening, evident by radiolucency beneath the anterior flange [6], when investigated further revealed failure at the cement-metal interface. However rather than the technique or implant used, it has been suggested that the likely critical factor in determining the long-term outcome appears to be linked to the postoperative high-flexion activities [6, 10, 11], where in the femoral component “loosened” group the mean maximum postoperative knee flexion angle was significantly larger. Further it was found that loosened femoral components migrated towards a more flexed position [6].

That such femoral component migration from loosening of the femoral component, associated with cement-metal failure, which in turn is linked to postoperative weight-bearing high-flexion activities, indicates a strong biomechanical causative factor to the problem. Studies from a mechanical standpoint have shown that the possible causes for the loosening could be due to the altered kinematics and increased bicondylar rollback in deep flexion [13], absence of femoral load sharing with the bone [14], and being less than ideal strength in the cement-metal interface [15]. These three mechanical factors may not only be interrelated, but also be individualised targets for finding a solution to the loosening problem. However it is important to note that the primary cause is yet to be determined and there still is further insight to be gained from seeking out what could be the mechanism that initiates the failure in the system.

In this paper, we report on a hypothesis on what could be an important mechanical factor that initiates anterior flange femoral component loosening. This hypothesis is based on (i) earlier studies carried out on normal knee deep flexion and squatting kinematics and kinetics, (ii) a finite element method (FEM) analysis, and (iii) known properties and strength of the implant-cement-bone interfaces.

## 2. Materials and Methods

### 2.1. Knee Joint Kinematics and Kinetics in Squatting

**Figure 1:**
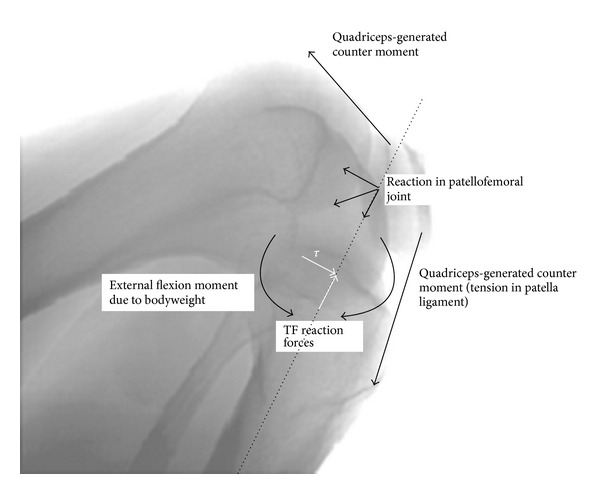
Schematic to illustrate how external flexion moments from reaction to body weight are balanced by internally generated moments from quadriceps activity. A backdrop of an X-ray image of a full squat is used as a reference. The reactions at the knee (illustrated as white arrows) show a compression and anterior-directed shear (*τ*).

**Figure 2:**
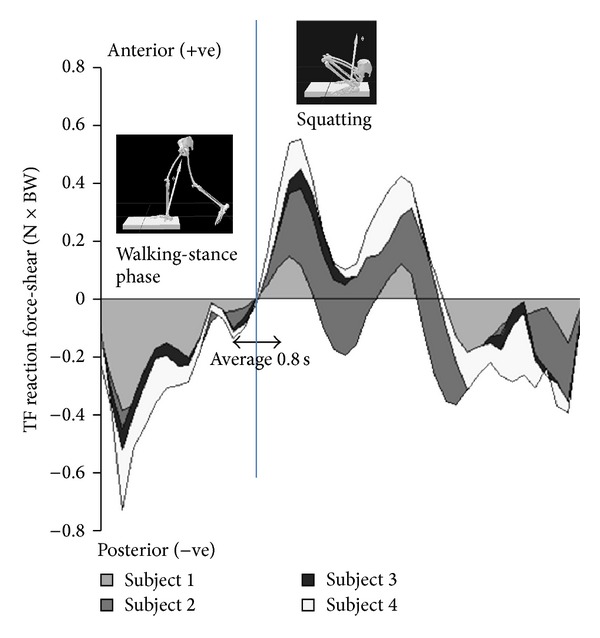
(Adapted from [12]), the reversal in shear reaction force in the tibiofemoral joint when the subject goes into a squat.

From gait studies it was found that in Asian-style squatting the knee flexes up to 150° and the tibiofemoral contact forces are as high as 3 to 4 times body weight [12, 16]. Translating the contact forces into stresses, in vitro studies on human cadaver knees showed that the peak pressures can be as high as 20 MPa as a result of the drastic reduction in contact area [17]. Coupled with high stresses, the external rotation of the femur about the tibia ranged from 10° to over 20° and from 2 mm to 4 mm posterior translation of the femur [17, 18], and it was found that in Japanese knees the posterior translation was twice that of Caucasian knees [18]. The extent of this movement was attributed to the differing surface contact morphology of the medial versus lateral tibial plateaus [19]. Importantly, the posterior translation of the femur over the tibia during the squat would result in an anterior-directed shear reaction force in the tibiofemoral joint (Figure 1). This kinematic feature of squatting, the anterior-directed shear, has received little attention in the literature, given that in walking and standing the shear reaction force is largely posterior-directed and in going into a squat the shear reverses into an anterior-directed one [12] (Figure 2). It is this mechanism that informs our hypothesis for femoral component loosening in the high flexion knee implants and provides the loading condition for our model.

### 2.2. Finite Element Analysis Model

**Figure 3:**
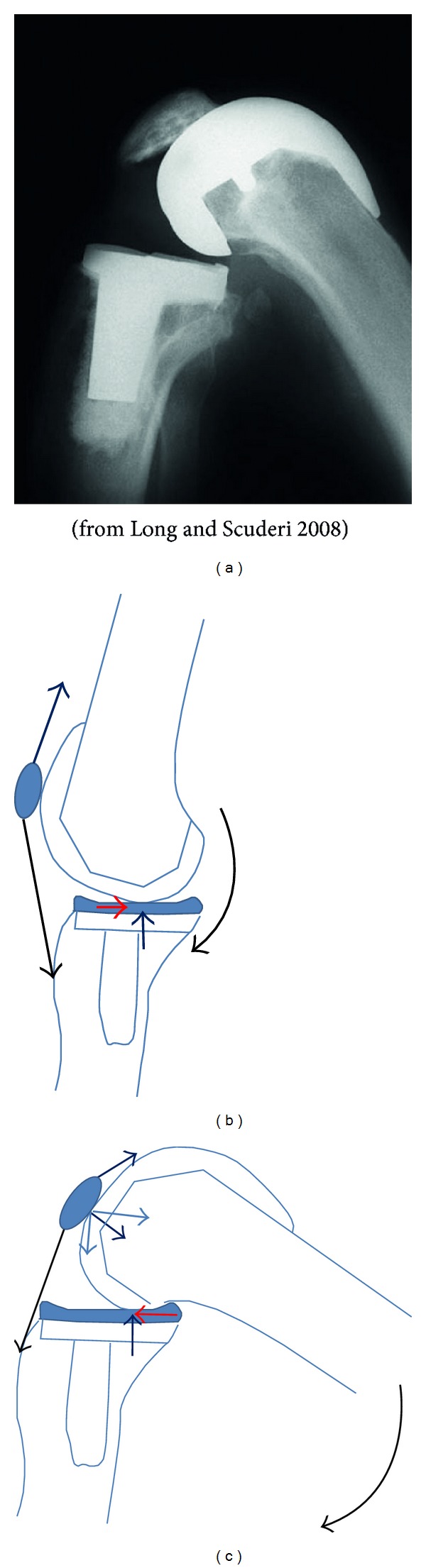
(a) Picture from [5], showing X-ray of implanted knee in deep flexion. (This picture has been rotated here to be consistent with the accompanying schematics.) (b) Force diagram to show the knee in extension. The short black and red arrows represent vertical and horizontal (shear) reactions in the tibiofemoral joint. The long arrows attached to the patella represent the force vectors of the patella tendon and ligament. The curved arrow represents the external moments acting on the knee, here in single-limb stance. (c) Force diagram showing the knee going into a squat. Note the reversal in shear (red arrow).

A finite element simulation was set up in Abaqus (Simulia) to evaluate the stress at the femoral bone-implant interface, specifically on the anterior flange. A generic knee model [20, 21] was developed and interfaced with a standard total knee replacement (TKR) configuration that consisted of cobalt chrome alloy femoral component, titanium tibial tray, polyethylene tibial insert, and polyethylene patellar button. The simulation was solved quasi-statically using Abaqus Standard with isotropic, linear elastic material properties. The Young's modulus of titanium, cobalt chrome alloy, polyethylene, and the cortical bone was set to 110 GPa, 220 GPa, 686 Mpa, and 15 GPa, respectively [22, 23]. The bone cement was assumed to be a rigid bond. Ligament and patellar tendon material stiffness properties were taken as 300 Nmm^−1^ [23]. We examined the change in anterior flange stress and strain due solely to the change in the externally applied shear stress reaction direction. We simulated two poses, the knee in (a) relative extension during the single-limb weight bearing mid-stance phase of the gait cycle and (b) deep squatting at 150° of flexion. The tibia was fixed in both cases and a vertical compressive force of 1750 N (2.5 BW) and a horizontal shear force of 450 N (0.68 BW) were applied. These forces were to create the reaction forces at the tibiofemoral joint described in an earlier study [12] (see Figure 2). In knee extension an anterior shear force was applied to the femur to give rise to a posterior shear reaction force at the tibiofemoral joint. In knee flexion it was* reversed* to give rise to an anterior shear reaction force (Figure 3).

### 2.3. Known Properties and Strength of the Implant-Cement-Bone Interfaces

There are two interfaces, the implant-cement and the cement-bone. These interfaces have different strengths and can be directly attributed to the type of surface involved. For example, surface finish in metals [24–26] and bone preparation for cement to engage cortical versus cancellous bone [15] all have an influence on the final achievable mechanical strength of the bond. The static* shear* strength of the implant-cement interface ranges from 3 MPa to 16 MPa, depending on the surface finish of the metal implant [24]. Tensile strength is found to be lower than shear [27] ranging between 0.58 and 6.67 MPa. For the cement-bone interface, depending on bone surface roughness, the shear strength involving cancellous bone is 3.85 MPa and the tensile strength is much lower at 1.79 MPa [15]. This finding is consistent with an earlier study showing that the cement-bone interface is weaker in tension than in shear [28]. Bone cement properties are also reported in terms of microstrain.

## 3. Results

**Figure 4:**
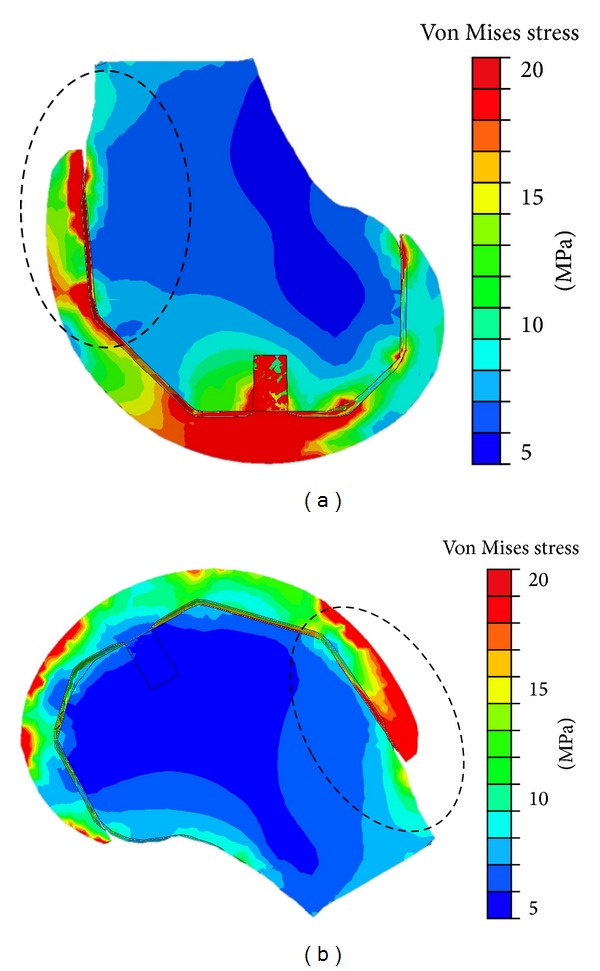
Sagittal cross-section of von Mises stress for (a) extended knee configuration and (b) deep knee flexion of 150°. Red is 20 MPa and dark blue is 5 MPa. The anterior flange region is circled.

To compare the von Mises stresses between the knee extension and knee flexion positions, a sagittal section of the model was made and the strain distribution was plotted in terms of a colour map (Figure 4). The model revealed that stresses ranged from 5 MPa (dark blue) to a maximum of 20 MPa (red) with bone showing low stresses near the anterior flange for both knee extension and flexion (Figure 4). It appeared that stress was primarily borne by the femoral component except where the implant interfaces with the bone at the site of tibial contact at the knee extension position.

**Figure 5:**
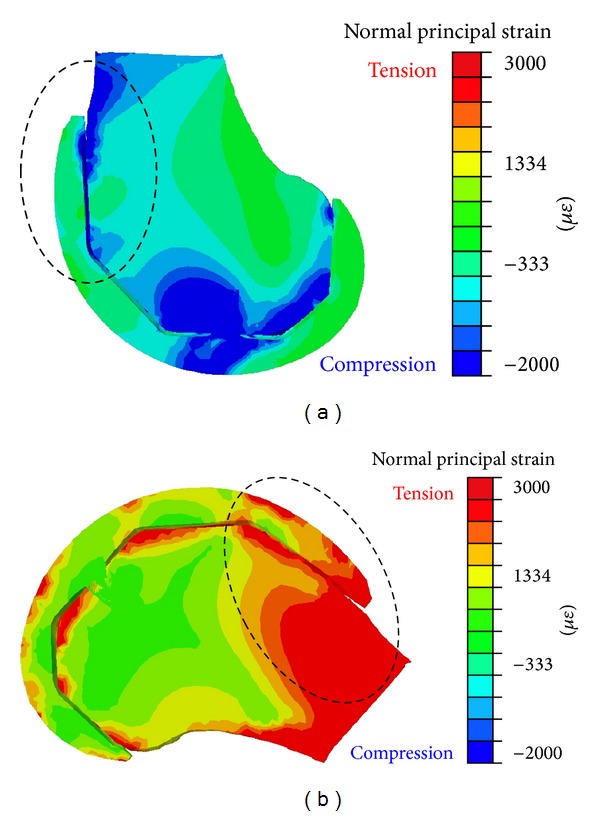
Sagittal cross-section of normal principal strains for (a) extended knee configuration and (b) deep knee flexion of 150°. Red is tension with a maximum of 3000 *με* and dark blue is compression at −2000 *με*. The anterior flange region is circled.

Of more interest were the principal strain values. To compare the maximum principal strains between the knee extension and knee flexion positions, a sagittal section of the model was made and the strain distribution was plotted in terms of a colour map (Figure 5). The strain ranged from a compressive normal strain of −2000 *με* (dark blue) in the knee extension position to a* tensile* normal strain of +3000 *με* (red) in full flexion. In knee extension the bulk of the bony strain was located near the inferior femoral condyles adjacent to the point of tibial contact. Also in this knee position, the maximum bone strain near the anterior flange occurred as two “hot-spots” (Figure 5(a), in blue within the dotted region) of compressive strain.

In contrast during deep knee flexion, and near the anterior flange, bone strain values were mostly tensile and spread over a much broader region along the length of the flange. The strain near the anterior flange during deep knee flexion exhibited a large mismatch between the bone and femoral component, where in the bone there is an overall larger region of high tensile strain compared to the femoral component (compare, e.g., regions of red in Figure 5(b)).

## 4. Discussion

**Figure 6:**
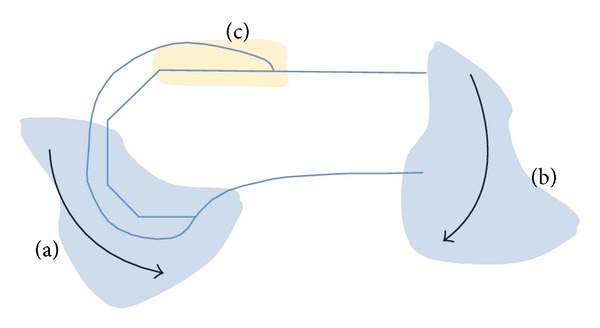
Schematic showing how the joint contact forces (vertical, shear, tibiofemoral, and patellofemoral) at (a) would be diametrically opposite the external body weight moment at (b) to create bending effects at (c).

For this study we only examined the influence of sagittal plane vertical and shear forces applied at the tibiofemoral contact, as the aim was to see the effect on the anterior flange region following a reversal of the shear direction. Any post-cam effects were not modeled and we argue that the transient anterior shear reaction that develops on going into a squat [12], meaning the femur tends to move posteriorly relative to the tibia, would not result in significant post-cam engagement. The effects of the femoral-patella contact or thigh-calf contact were also not modeled and it is without doubt that these would be important for any full knee flexion loading simulation [29]. However, these effects may not influence this study's focus on the anterior flange region for the following reasons. Firstly, in deep flexion, it is very likely that the femoral-patella contact, being very proximal to the joint line (Figure 3(a)), and from observation of the force diagram (Figure 3(c)), will result in moments that tend to bend the femur with respect to the femoral implant (Figure 6). Such bending stresses will thus tend to add to the bone bending stresses in the femur, confirmed by the increased tensile strain (shown in red in Figure 3(b)), in the distal femoral shaft in the knee flexion position. Secondly, the application of the shear reversal follows the gait data obtained previously [12] that was shown to happen prior to the rest phase in squat when the thigh contacts the calf. Hence, the simulation used in the present study, albeit simple in its approach, we argue, is valid for investigating a direct cause-effect relationship between the applied shear at the tibiofemoral joint and the resulting stresses and strain at the anterior flange.

The effects following the shear reversal, when the knee is in the flexed position, are larger von Mises stresses at the anterior flange region. The von Mises stresses provide a more realistic means of predicting failure criteria based on the combined effects of multidirectional stresses, as opposed to a unidirectional one. Therefore that the range of values for the ultimate stress for the implant-cement-bone interfaces, as described earlier in the methods section, is below the approximately 20 MPa high von Mises stress at the anterior flange region for the knee flexed position indicates that tibiofemoral shear reversal may be a causative factor for problems at this region in high flexion cases.

In addition, the tensile strains at the anterior flange for the knee flexed position, compared with the extension position where the strains in the same region were mostly compressive, provide additional insight into how the shear reversal effect may be detrimental to the implanted joint. In terms of maximum strains, the 3000 *με* measured in the present model simulation of knee deep flexion is well below the failure range between 15,000 *με* and 250,000 *με* [30], meaning that the strain measured may not be of concern in terms of a single cycle static strain. However, taken into context of cyclic loading and creep, it would be a different matter. Previous experiments on bone cement strength have shown that 1000 *με* results in failure after 10 million cycles [31]. The question then would be if such a lifespan could be reduced by (1) periodic interjections of 3000 *με* tensile loading on going into and coming up from a squat, (2) possible sustained loading during the squatting phase [32] resulting in bone cement creep effects [33–35], and (3) bone cement being weakest in tension [15, 28, 36].

Furthermore, from the simulation (Figure 5) comparing extension to deep flexion, the high strain gradient from compression to tension, which when considering would be occurring rapidly [12] and bone cement as being a viscoelasticmaterial, may make the material increasingly strain limiting [37, 38] and susceptible to cracking. In addition the large mismatch in strain magnitude and strain pattern at the bone-implant anterior flange interface for deep flexion may lead to implant loosening due to failure of the bone cement in these regions. To note as well is the increased bone strain at levels of 3000 *με* for deep knee flexion, in the distal femur near the anterior shaft all along to beneath the flange, which may lead to increased bone damage. Importantly, at these levels, damaged bone may be resorbed [39], which may then predispose the implant to loosening.

## 5. Conclusions

To conclude, we deduce that the rapid reversal of the tibiofemoral shear reaction, from going into and coming up from a squat, constitutes a significant biomechanical factor for the possible failure mechanisms reported involving high flexion knee femoral component loosening. This rapid shear reversal, if incorporated into simulated loadings of implants in in vitro materials testing systems, may provide further insight into mechanisms of implant failure typically attributed to deep flexion knee activity.
